# Prevalence and Predictors of Cancellation of Elective Surgical Procedures at a Tertiary Hospital in Uganda: A Cross-Sectional Study

**DOI:** 10.1155/2020/1464098

**Published:** 2020-03-19

**Authors:** Alfred Ogwal, Felix Oyania, Emmanuel Nkonge, Timothy Makumbi, Moses Galukande

**Affiliations:** ^1^Department of Surgery, St Joseph's Hospital, Maracha, P.O. Box 59, Arua, Uganda; ^2^Department of Surgery, Mbarara Regional Referral Hospital, P.O. Box 1410, Mbarara, Uganda; ^3^Department of Surgery, Kitovu Hospital, P.O. Box 413, Masaka, Uganda; ^4^Department of Surgery, College of Health Sciences, Makerere University, P.O. Box 7062, Kampala, Uganda

## Abstract

*Introduction*. The cancellation of elective procedures has been shown to waste resources and to have the potential to increase morbidity and mortality among patients. This study aimed to determine the prevalence of the cancellation of elective surgical procedures and to identify the factors associated with these cancellations at Mulago Hospital, a large public hospital in Kampala, Uganda.

**Methods:**

A cross-sectional study was conducted from January 10, 2018, to February 20, 2018. We recruited patients of all ages who were admitted to surgical wards and scheduled for elective surgery. Data on patients' demographic characteristics and diagnosis, as well as the specialty of the surgery, the planned procedure, the specific operating theatre, cancellation, and the reasons for cancellation were extracted and analyzed using logistic regression.

**Results:**

Of a total of 400 cases, 115 procedures were canceled—a cancellation prevalence of 28.8%. Orthopedic surgery had the highest cancellation rate, at 40.9% (*n* = 47). Facility-related factors were responsible for 67.8% of all cancellations. The most common reason for cancellation was insufficient time in the theatre to complete the procedure on the scheduled day. No procedures were canceled because of a lack of intensive care unit beds. There was a significant association between surgical specialty and cancellation (*P* < 0.05) at multivariate analysis.

**Conclusion:**

The prevalence of cancellation of elective surgical procedures at Mulago Hospital was 28.8%, with orthopedic surgery having the highest cancellation rate. Two-thirds of the factors causing cancellations were facility-related, and more than 50% of all cancellations were potentially preventable. Quality-improvement strategies are necessary in the specialties that are susceptible to procedure cancellation because of facility factors.

## 1. Introduction

An elective surgical procedure is said to be canceled when a patient's name appears on the list for surgical operations, but the operation is not performed on the scheduled date [1]. Previous work has reported cancellation rates for elective surgeries to range from 1.96% to 49% [2, 3]. In Western countries, about 20% of elective surgeries are canceled on the day of surgery [4]. However, in low-income countries, cancellation rates are as high as 48.5% [3]. These cancellations are associated with wide-ranging clinical, psychological, and economic consequences to the patients, hospitals, and health care providers, although patients are the most affected [5]. The causes of cancellation of elective surgical procedures are multifactorial and tend to vary across health facilities and countries [6]. Notably, most cancellations of elective surgical procedures occur because of various modifiable reasons [7, 8]. Some previous studies have grouped the reasons for cancellation into relatively broad categories, and others have simply listed causes without grouping them [9].

In Uganda, the cancellation of elective surgical operations is very common in routine clinical practice. However, no study has explored the reasons why these procedures are not performed on the scheduled dates. Moreover, the true prevalence of cancellation is unknown. Previous studies conducted in low-income and developed countries have produced a strong body of evidence suggesting that the burden of these cancellations is excessively high in developing countries. Therefore, the present study aimed to establish the prevalence of cancellation of elective surgical procedures and to identify the factors associated with these cancellations among patients in surgical wards at Mulago Hospital in Uganda.

## 2. Materials and Methods

### 2.1. Study Setting

This study was conducted at Mulago National Referral and Teaching Hospital, a tertiary teaching hospital affiliated with Makerere University College of Health Sciences with a capacity of 1,790 beds. This study involved the surgical units under the Directorate of Surgery that were based in the Old Mulago complex at the time of the research: general surgery; breast and endocrine surgery; ear, nose, and throat surgery; pediatric surgery; neurosurgery; cardiothoracic surgery; orthopedic surgery; urological surgery; and oromaxillofacial surgery. At the time of the study, the hospital was undergoing a major renovation of the New Mulago complex, which normally houses the departments of general surgery, breast and endocrine surgery, urological surgery, neurosurgery, and pediatric surgery. During this renovation, these departments were temporarily moved to Old Mulago.

At Old Mulago Hospital, elective surgeries are currently performed in seven different operating theatres, all of which were functional during the entire study period. The hospital's elective surgical lists are prepared by intern doctors and surgical residents in consultation with the surgeons affiliated with the different surgical specialties. Prior to ward admission, all patients attend a surgical outpatient clinic, where they are registered and given a date to return to check whether there is bed space in the inpatient ward. In the various inpatient wards, patients are supposed to be fully prepared and then scheduled for elective procedures, and the responsible surgeon in each ward signs off on the surgical list for that ward. The signed list is then taken to the theatre by 2:00 PM on the day prior to the procedure date. Elective procedures begin at 8:00 AM and are required to be finished by 5:00 PM. Each day at about 4:30 PM (sometimes earlier), procedures with no reasonable prospect of being completed by 5:00 PM are canceled.

### 2.2. Study Design and Procedure

This was a cross-sectional study conducted from January 10 to February 20, 2018. A canceled procedure was defined as a patient's name appearing on the list for surgical operations but the operation not being performed on the scheduled date. Patients scheduled for surgical procedures were recruited into this study by the principal investigator and research assistants on the day prior to their operation. Patients with multiple cancellations were excluded from the study. Data were collected on demographic characteristics, diagnosis, surgical specialty, planned procedure, assigned theatre, cancellation, and reasons for cancellation. In terms of reasons for cancellation, medical team members were able to select multiple reasons from a provided list, and they were also invited to add reasons that were not on the list. The theatre nurses on duty provided consent and were interviewed by the study team to complete a questionnaire collecting data on these items. Other staff members working in the operating theatres and surgical teams were interviewed when clarification was required.

### 2.3. Statistical Analysis

Data from the completed questionnaires were double entered into Epidata, Version 3.1, with programmed quality-control checks. After editing, the data were exported to Stata 13.0 for analysis. For the presentation of descriptive statistics, categorical variables are summarized using percentages, and proportions and means, standard deviations, medians, and interquartile ranges are used for continuous variables. The overall prevalence of cancellation was calculated by dividing the total number of cancellations by the total number of operations scheduled. A binary logistic regression model was used to identify factors that were statistically associated with the cancellation of elective procedures. Odds ratios (ORs) and 95% confidence intervals (CIs) of cancellation were estimated for the different specialties and specific theatres. The level of significance was set at *P* < 0.05.

### 2.4. Ethical Considerations

This study was conducted with the approval of the School of Medicine Research and Ethics Committee of Makerere University College of Health Sciences (#REC REF 2018-007). Patients and/or their next of kin provided consent for all included patients' participation in this study. For minors, consent was obtained from emancipated minors, and assent was obtained from other children.

## 3. Results and Discussion

### 3.1. Results

Baseline characteristics of 400 patients scheduled for elective procedures are provided in Table 1.

In total, 400 participants were recruited, with a male-to-female ratio of 2.1 : 1. The most frequent surgical specialty represented was orthopedics (41.5%). Among the recruited patients, there were 108 (27.0%) children aged 0–9 years, 57 (14.2%) adolescents aged 10–19 years, 164 (41.0%) adults aged 20–49 years, and 71 (17.8%) adults aged ≥50 years. The main operating theatre had the highest number of procedures schedule at 30.3% and Uganda Heart Institute theatre had the least number, representing 5.3% of the total procedures scheduled (Table 1).

Many reasons were reported to be contributing to the cancellation of elective surgical procedures at Mulago hospital. These included insufficient time in the theatre to complete the procedure by the required time (5:00 PM) (*n* = 44 cases), no anesthetist being available (*n* = 21 cases), and emergency cases being prioritized (*n* = 9 cases) (Table 2).

**Figure 1:**
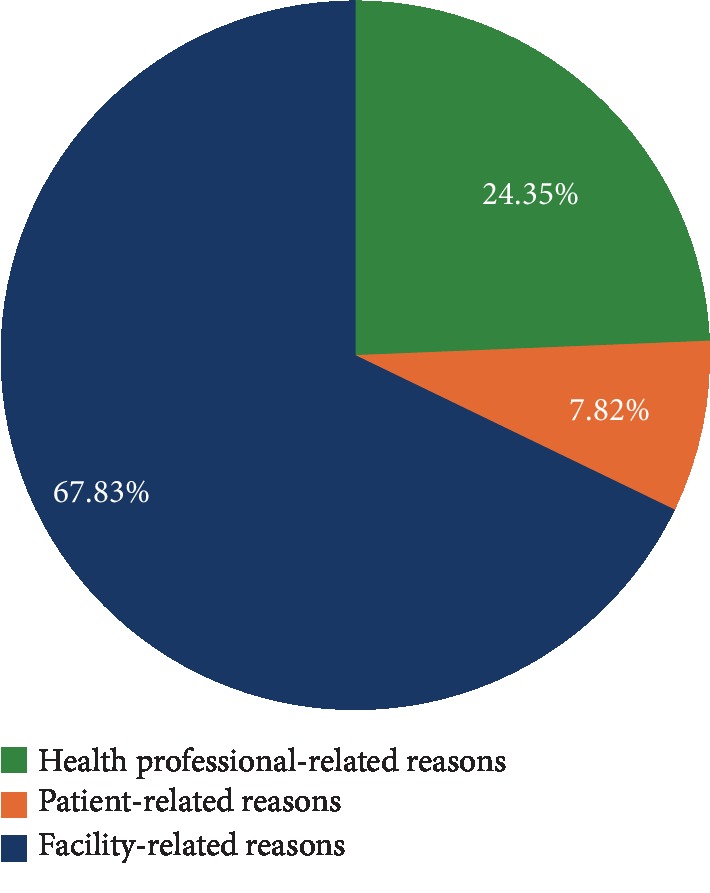
Pie chart showing the percentage distribution of factors contributing to cancellation of elective surgeries by category.

Facility-related factors were responsible for more than two-thirds of the cancellations at Mulago Hospital (Figure 1).

The observed prevalence of cancellation of elective surgical procedures was 28.8% (*n* = 115). The prevalence of cancellation was approximately 50.4% (*n* = 58) among adults aged 20–49 years and 10.5% (*n* = 12) among adolescents aged 10–19 years. Of the examined surgical specialties, the cancellation prevalence was highest in orthopedic surgery, at 40.9% (*n* = 47), closely followed by neurosurgery at 25.2% (*n* = 29) and cardiothoracic surgery having the least cancellation rate at 0.9% (*n* = 1) (Table 3).

In the bivariate analysis, patient's age, surgical specialty of the operation, and operating theatre each had a *P* value of less than 0.2 (Table 3). These variables were therefore selected for consideration in the multivariate analysis.

The multivariate analysis showed that surgical specialty was significantly associated with the cancellation of elective surgical procedures. The odds of an elective surgical procedure being canceled were about four times as high for the neurosurgery specialty than for the reference group of breast and endocrine surgery (adjusted odds ratio = 4.13, 95% confidence interval: 1.19–14.33, *P* value = 0.026) (Table 4).

## 4. Discussion

There is no consensus on the maximum acceptable case cancellation rate for efficient operating theatres, but rates under 5% are generally recommended [10]. Our finding that 28.8% of elective surgical procedures were cancelled on the day of surgery is similar to the results of Ebirim et al.'s finding of a cancellation rate of 28% in Nigeria [11]. However, our result is higher than cancellation rates shown in other studies [12, 13]. The relatively high cancellation rate in our study may be explained by the fact that we investigated all possible factors associated with the cancellation of elective surgical operations, ranging from patient-related factors such as abnormal preoperative tests to facility-related factors such as insufficient time in the operating theatre to complete the procedure on the scheduled day. The ongoing renovations and associated internal readjustment and relocation of the theatre spaces may also explain the high cancellation rate found in the present study.

Previous studies have observed different rates of cancellation depending on the how they defined the cancellation of elective operations, with reported cancellation rates ranging from 1.96% to 49%) [2, 3]. Findings on the rate of cancellation also vary widely by study design; hospital type, level, and capacity; patient type (inpatients vs. outpatients); and surgical specialty [14–19]. In the present study, orthopedic surgery had the highest cancellation rate (40.9%) of the studied surgical specialties. This high rate may be a reflection of the fact that this department had a longer theatre list compared with all the other specialties, thus increasing the likelihood of cancellation. Neurosurgery had the second highest cancellation rate. This is probably because the neurosurgery department usually had a late start for the first surgical procedure each day. The high rate of cancellation within this specialty may also be explained by the fact that this department was not working on neurotrauma emergencies in emergency department when this study was conducted. Thus, these emergency cases were added to the elective list and were frequently given priority over elective procedures. We found that the narrow specialty of cardiothoracic surgery had the lowest cancellation rate, at 0.9%. This may be because approximately two to three cases under this specialty are scheduled per operating theatre each day. However, it is also possible that specialties with lower cancellation rates may have had fewer operating days per week, compared with specialties with higher cancellation rates.

Facility-related reasons were the most commonly listed factors explaining the cancellation of elective surgical procedures. This finding is in line with previous studies conducted in Tanzania and Nigeria [19, 20]. More specifically, the most common reason listed under this category was insufficient time to complete the procedure in its assigned operating theatre before the end of the day. This typically occurred because surgeries performed earlier in the day lasted longer than anticipated. In specialties where facility-related factors were a common reason for cancellation, it may be that lists were intentionally overbooked with the aim of increasing the likelihood that all procedures could be completed. Cancellations caused by these facility-related reasons are potentially preventable if due diligence is exercised throughout the process of preparing the surgical list. Pandit et al. [6] noted that overbooking surgical lists, which was responsible for half of the observed surgical cancellations in their study, was done because of waiting-list pressures and to avoid the perception that the surgical team was not hard-working.

Strategies such as reducing operating theatre turnover time, starting the first procedure on time, setting up anesthesia equipment and any other auxiliary equipment ahead of time, and gathering supplies in a timely fashion can be implemented in parallel to increase operating theatre efficiency. At Mulago hospital, the cause of time running out in an operating theatre is multifactorial. The number of occurrences of this can be reduced through cooperation across all disciplines. Offering “overtime” payments may also provide an incentive to prevent surgical cancellation, but this strategy may lead to very high overall hospital costs if overbooking is common. Routine and fair estimations of procedure durations may also be helpful in improving operating theatre usage.

Blood shortages were another reason contributing to elective procedure cancellation in the present study. An earlier study by Kajja and Sibinga [21] also noted that the lack of blood was a major cause of delayed scheduling of elective surgery. In our study, blood shortages were mainly observed in the specialties of pediatrics, urology, and orthopedics. These shortages were likely exacerbated by financial constraints at Uganda Blood Transfusion Services, as reported by Okiror in *the Guardian* newspaper [22]. Additionally, it is possible that some surgical units did not reserve blood for their patients prior to the day of the surgery.

No procedures at Mulago Hospital were canceled during the study period because of insufficient intensive care unit (ICU) beds. This finding differs from the results of a 2012 study conducted in Hong Kong, where the lack of ICU beds explained 14% of surgical cancellations [22, 23]. At Mulago Hospital, ICU beds are requested prior to scheduling an elective procedure. If the ICU team reports that no bed is available, the procedure automatically does not appear on a particular theatre list for that day. However, it may also be possible that, for various reasons, some narrow specialties are not scheduling highly complex surgeries that require postoperative cardiac or respiratory support.

The absence of anesthetists was found to be a problem in this study. Similar observations have been reported in other studies [15, 19, 24, 25]. Staff shortages and lack of health resources are common problems in most hospitals in sub-Saharan Africa [15]. At times, anesthetists were unavailable because they were attending a workshop or a conference. Considering the resource limitations in this hospital context, a redistribution of available staff members and other appropriate arrangements may be made to internally cover a staff member's annual or study leave.

Other health professional-related reasons, such as a change of diagnosis by the surgeon or a change in the management plan, are preventable. Adequate clinical assessment by all members of the surgical team, including senior surgeons, can mitigate obvious omissions by junior staff members such as the surgical residents closely involved in generating the elective surgical lists.

Patient-related factors were the least commonly listed category of reasons for procedure cancellation. Cancellations because of patient-related reasons were mainly because of abnormal preoperative tests, especially low levels of hemoglobin. The situation was compounded by the shortage of blood for perioperative and postoperative blood transfusion. This result differed from the findings reported by Gajida et al. [3], who found that patients leaving the hospital before surgery and patients' lack of funds to pay for the surgery were the main patient-related reasons for cancellation. This difference may be explained by the fact that the hospital in the present study is a public no-fee hospital, whereas Gajida et al.'s study was conducted at a federal tertiary hospital where patients pay for service. Our findings also differ from those studies conducted in Australia [26] and the United Kingdom [27], possibly because the patients in these previous studies paid for surgical services, either out-of-pocket or through an insurance scheme.

The main limitation of the present study was that we did not include patients who had multiple cancellations. This omission may have led to some degree of underestimation of the cancellation rate because some patients do experience multiple cancellations. However, we believe that our results, which are based on real-world clinical data from a large hospital, will be broadly useful to health care practitioners and hospital administrators throughout Uganda and in other similar contexts.

## 5. Conclusions

The prevalence of cancellation of elective surgical procedures at Mulago Hospital was 28.8%, with the highest cancellation rate found in the specialty of orthopedic surgery. Two-thirds of the procedure cancellations were caused by facility-related reasons, with more than 50% of the observed cancellations being potentially preventable. Quality-improvement strategies are necessary in surgical specialties that are susceptible to procedure cancellations caused by facility factors.

## Figures and Tables

**Table 1:** Baseline characteristics of 400 patients scheduled for elective procedures in surgical wards at Mulago Hospital, Uganda.

Variable	Frequency	Percentage (%)
*Patient's age (years)*		
0-9	108	27.0
10–19	57	14.2
20–49	164	41.0
≥50	71	17.8

*Patient's sex*		
Female	131	32.7
Male	269	67.3

*Surgical specialty*		
Breast and endocrine surgery	18	4.5
Cardiothoracic surgery	23	5.8
Ear, nose, and throat surgery	26	6.5
General surgery	24	6.0
Neurosurgery	62	15.5
Oromaxillofacial surgery	7	1.8
Orthopedic surgery	166	41.5
Pediatric surgery	38	9.5
Urological surgery	36	9.0

*Operating theatre*		
Main operating theatre	121	30.3
Naguru pediatric theatre^a^	44	11.0
Spine theatre	43	10.8
Trauma theatre	52	13.0
Uganda Cancer Institute theatre	30	7.5
Uganda Heart Institute theatre	21	5.3
Ward 7 theatre^b^	89	22.3

^a^Naguru Hospital is a general hospital where pediatric surgical procedures performed by Mulago Hospital staff were conducted at the time of the study. ^b^Ward 7 is an orthopedic ward at Mulago Hospital with an attached operating theatre.

**Table 2:** Reasons for cancellation of elective procedures in surgical wards at Mulago Hospital, Uganda.

Variable	Frequency (*n*)
*Health professional-related reasons*	
Surgeon with required expertise not available	2
Surgeon changed patient's diagnosis	2
Anesthetist not available	21
Theatre nurse not available	6
Surgeon changed patient's management plan	3

*Patient-related reasons*	
Patient did not follow preoperative instructions	3
Patient declined to undergo the procedure	1
Patient had abnormal preoperative tests	7
Patient found to have undiagnosed congenital heart disease	1
Patient left the hospital	1

*Facility-related reasons*	
Emergency case prioritized	9
Sterile instruments or consumables not available	5
Power outage before the procedure	2
Oxygen not available in the theatre	2
Required equipment broken or unavailable	6
Insufficient time in the theatre to complete the procedure by the required time (5:00 PM)	44
Impromptu theatre fumigation	4
Shortage of blood	6
No ambulance available for patient transfer to postoperative ward	2
No intensive care unit beds available	0

**Table 3:** Bivariate analysis of factors associated with cancellation of elective procedures in surgical wards at Mulago Hospital, Uganda.

Variable	Cancellation of surgery		Crude odds ratio	95% confidence interval	*P*-value
	Yes	No			
*Patient's age (years)*					
0–9	22 (19.1)	86 (30.2)	1		
10–19	12 (10.5)	45 (15.8)	1.04	0.47–2.30	0.918
20–49	58 (50.4)	106 (37.2)	2.14	1.21–3.77	0.009
≥50	23 (20.0)	48 (16.8)	1.87	0.95–3.71	0.072

*Patient's sex*					
Female	39 (33.9)	92 (32.3)	1		
Male	76 (66.1)	193 (67.7)	0.93	0.59–1.47	0.753

*Surgical specialty*					
Breast and endocrine surgery	4 (3.5)	14 (4.9)	1		
Cardiothoracic surgery	1 (0.9)	22 (7.7)	0.16	0.02–1.57	0.116
Ear, nose, and throat surgery	3 (2.6)	23 (8.1)	0.46	0.09–2.35	0.348
General surgery	6 (5.2)	18 (6.3)	1.17	0.28–4.94	0.834
Neurosurgery	29 (25.2)	33 (11.6)	3.08	0.91–10.40	0.071
Oromaxillofacial surgery	2 (1.7)	5 (1.8)	1.4	0.19–10.15	0.739
Orthopedic surgery	47 (40.9)	119 (41.7)	1.38	0.43–4.12	0.585
Pediatric surgery	8 (6.9)	30 (10.5)	0.93	0.24–3.62	0.921
Urological surgery	15 (13.1)	21 (7.4)	2.5	0.68–9.11	0.165

*Operating theatre*					
Main operating theatre	41 (35.7)	80 (28.1)	1		
Naguru pediatric theatre^a^	9 (7.8)	35 (12.3)	0.5	0.22–1.14	0.101
Spine theatre	13 (11.3)	30 (10.5)	0.85	0.40–1.79	0.662
Trauma theatre	12 (10.4)	40 (14.0)	0.59	0.28–1.24	0.16
Uganda Heart Institute theatre^b^	0 (0.00)	21 (7.4)	—	—	—
Ward 7 theatre^c^	26 (22.6)	63 (22.1)	0.81	0.44–1.45	0.473
Uganda Cancer Institute theatre	14 (12.2)	16 (5.6)	1.71	0.76–3.84	0.196

^a^Naguru Hospital is a general hospital where pediatric surgical procedures performed by Mulago Hospital staff were conducted at the time of the study. ^b^There were no cancellations in the Uganda Heart Institute theatre, so no bivariate results could be estimated. ^c^Ward 7 is an orthopedic ward at Mulago Hospital with an attached operating theatre.

**Table 4:** Multivariate logistic regression analysis of factors associated with cancellation of elective procedures in surgical wards at Mulago Hospital, Uganda.

Variable	Crude odds ratio	Adjusted odds ratio	95% confidence interval	*P* value
*Patient's age (years)*				
0-9	1	1		
10–19	1.04	0.87	0.37–2.07	0.752
20–49	2.14	1.92	0.96–3.85	0.064
≥50	1.87	2.08	0.92–4.67	0.077

*Surgical specialty*				
Breast and endocrine surgery	1	1		
Cardiothoracic surgery	0.16	0.23	0.02–2.34	0.212
Ear, nose, and throat surgery	0.46	0.68	0.13–3.59	0.646
General surgery	1.17	1.17	0.28–4.96	0.833

*Neurosurgery*	**3.08**	**4.13**	**1.19–14.33**	**0.026**
Oromaxillofacial surgery	1.4	1.53	0.21–11.18	0.675
Orthopedic surgery	1.38	1.76	0.54–5.69	0.346
Pediatric surgery	0.93	1.87	0.42–8.40	0.413
Urological surgery	2.5	3.31	0.89–12.33	0.074

Bold typeface indicates statistical significance at *P* < 0.05.

## Data Availability

The data used to support this study are available from the corresponding author upon reasonable request.
